# The mobilization and transport of newly fixed carbon are driven by plant water use in an experimental rainforest under drought

**DOI:** 10.1093/jxb/erae030

**Published:** 2024-01-25

**Authors:** Jianbei Huang, S Nemiah Ladd, Johannes Ingrisch, Angelika Kübert, Laura K Meredith, Joost van Haren, Ines Bamberger, L Erik Daber, Kathrin Kühnhammer, Kinzie Bailey, Jia Hu, Jane Fudyma, Lingling Shi, Michaela A Dippold, Kathiravan Meeran, Luke Miller, Michael J O’Brien, Hui Yang, David Herrera-Ramírez, Henrik Hartmann, Susan Trumbore, Michael Bahn, Christiane Werner, Marco M Lehmann

**Affiliations:** Max Planck Institute for Biogeochemistry, D-07745 Jena, Germany; Ecosystem Physiology, Albert-Ludwig-University of Freiburg, Freiburg, Germany; Department of Environmental Sciences, University of Basel, Bernoullistrasse 30, 4056 Basel, Switzerland; Ecosystem Physiology, Albert-Ludwig-University of Freiburg, Freiburg, Germany; Department of Ecology, University of Innsbruck, Sternwartestr 15, 6020 Innsbruck, Austria; Ecosystem Physiology, Albert-Ludwig-University of Freiburg, Freiburg, Germany; School of Natural Resources and the Environment, University of Arizona, 1064 E. Lowell St., Tucson, AZ 85721, USA; Biosphere 2, University of Arizona, 32540 S. Biosphere Rd, Oracle, AZ 85739, USA; Biosphere 2, University of Arizona, 32540 S. Biosphere Rd, Oracle, AZ 85739, USA; Honors College, University of Arizona, 1101 East Mabel Street, Tucson, AZ 85719, USA; Ecosystem Physiology, Albert-Ludwig-University of Freiburg, Freiburg, Germany; Atmospheric Chemistry Group, University of Bayreuth (BayCEER), Germany; Ecosystem Physiology, Albert-Ludwig-University of Freiburg, Freiburg, Germany; Ecosystem Physiology, Albert-Ludwig-University of Freiburg, Freiburg, Germany; School of Natural Resources and the Environment, University of Arizona, 1064 E. Lowell St., Tucson, AZ 85721, USA; School of Natural Resources and the Environment, University of Arizona, 1064 E. Lowell St., Tucson, AZ 85721, USA; Department of Environmental Science, University of Arizona, Tucson, AZ, USA; Department of Land, Air, and Water Resources, University of California, Davis, CA, USA; Biogeochemistry of Agroecosystems, University of Göttingen, Göttingen, Germany; Geo-Biosphere Interactions, University of Tuebingen, Tuebingen, Germany; Biogeochemistry of Agroecosystems, University of Göttingen, Göttingen, Germany; Geo-Biosphere Interactions, University of Tuebingen, Tuebingen, Germany; Department of Ecology, University of Innsbruck, Sternwartestr 15, 6020 Innsbruck, Austria; Biosphere 2, University of Arizona, 32540 S. Biosphere Rd, Oracle, AZ 85739, USA; Estación Experimental de Zonas Áridas, Consejo Superior de Investigaciones Científicas, Almería, Spain; Max Planck Institute for Biogeochemistry, D-07745 Jena, Germany; Max Planck Institute for Biogeochemistry, D-07745 Jena, Germany; Max Planck Institute for Biogeochemistry, D-07745 Jena, Germany; Institute for Forest Protection, Julius Kühn Institute (JKI) - Federal Research Centre for Cultivated Plants, Erwin-Baur-Straße 27, D-06484 Quedlinburg, Germany; Max Planck Institute for Biogeochemistry, D-07745 Jena, Germany; Department of Ecology, University of Innsbruck, Sternwartestr 15, 6020 Innsbruck, Austria; Ecosystem Physiology, Albert-Ludwig-University of Freiburg, Freiburg, Germany; Swiss Federal Research Institute WSL, 8903 Birmensdorf, Switzerland; INRAE-Montpellier, France

**Keywords:** Carbon allocation, drought and climate change, ecosystem isotopic labeling, non-structural carbohydrate storage, plant hydraulics, tropical forests

## Abstract

Non-structural carbohydrates (NSCs) are building blocks for biomass and fuel metabolic processes. However, it remains unclear how tropical forests mobilize, export, and transport NSCs to cope with extreme droughts. We combined drought manipulation and ecosystem ^13^CO_2_ pulse-labeling in an enclosed rainforest at Biosphere 2, assessed changes in NSCs, and traced newly assimilated carbohydrates in plant species with diverse hydraulic traits and canopy positions. We show that drought caused a depletion of leaf starch reserves and slowed export and transport of newly assimilated carbohydrates below ground. Drought effects were more pronounced in conservative canopy trees with limited supply of new photosynthates and relatively constant water status than in those with continual photosynthetic supply and deteriorated water status. We provide experimental evidence that local utilization, export, and transport of newly assimilated carbon are closely coupled with plant water use in canopy trees. We highlight that these processes are critical for understanding and predicting tree resistance and ecosystem fluxes in tropical forest under drought.

## Teaser

The mechanisms underlying drought-induced changes in allocation of newly assimilated carbohydrates differ between water-saving and water-spending canopy trees, revealed by an ecosystem-scale ^13^ CO_2_ pulse-labeling experiment in the Biosphere 2 rainforest.

## Introduction

Tropical forests play an important role in the global carbon cycle by contributing substantially to terrestrial carbon sequestration (Beer *et al.*, 2010; Piao *et al.*, 2020). However, climate extremes, such as drought and heat waves, are occurring more frequently in tropical regions under ongoing climate change (Boisier *et al.*, 2015; Duffy *et al.*, 2015; Anderegg *et al.*, 2022). Extreme drought events can greatly reduce the ability of tropical forests to assimilate and partition carbon among different sinks (Brienen *et al.*, 2015; Brando *et al.*, 2019; Hubau *et al.*, 2020). While this may have severe consequences for the global carbon cycle and climate feedbacks, predictions of how drought impacts tropical forest dynamics still remain highly uncertain, in part due to our poor understanding and model representation of the key physiological processes underlying tropical plant responses to drought (Huntingford *et al.*, 2013).

Non-structural carbohydrates (NSCs), consisting primarily of soluble sugars and starch, are a critical component of tree acclimation and adaptation to environmental stress (Chapin *et al.*, 1990; Hartmann and Trumbore, 2016). NSCs are the major carbon storage pool that buffers the carbon imbalance between supply via photosynthesis and demands for growth and respiration under drought. Carbohydrate allocation from source to sink organs also determines whole-plant carbon allocation patterns (Sala *et al.*, 2010; Savage *et al.*, 2016). Drought theoretically limits carbohydrate export from the leaves and phloem transport by reducing leaf carbohydrate availability and the phloem turgor pressure gradient (Sala *et al.*, 2010; Salmon *et al.*, 2019). This has been demonstrated in drought manipulation and isotopic labeling experiments, which showed that drought increased the mean residence time (MRT) of recently assimilated carbohydrates labeled with ^13^C in leaves and led to a delay in the appearance of the ^13^C-label in phloem and/or roots (Salmon *et al.*, 2019). However, most such labeling experiments have been conducted on a single temperate species such as beech (Ruehr *et al.*, 2009; Hagedorn *et al.*, 2016; Dannoura *et al.*, 2018; Hesse *et al.*, 2018), and there is little experimental evidence for tropical forests with diverse plant species.

A comprehensive assessment of drought impacts on NSC dynamics in tropical forests requires consideration of the diverse plant functional types that represent differences in hydraulic strategy and canopy position. For example, some species tend to close stomata to maintain leaf water status and hydraulic integrity at the cost of reduced supply of photosynthates under drought, while others may tend to open stomata for photosynthetic gain at the cost of increased water loss. Tall canopy trees grown in high light are expected to have greater photosynthetic supply and faster processing of carbohydrates to enhance their ability to exploit resources compared with understorey plants grown in low light (Reich, 2014; Poorter *et al.*, 2019). Despite having larger root systems, tall canopy trees have higher evaporative demand and face greater hydraulic challenges than small understorey plants, and thus are often more susceptible to drought (Bennett *et al.*, 2015; Liu *et al.*, 2021). Therefore, plant water use strategies and canopy positions may determine the extent to which drought affects carbohydrate assimilation and allocation. It remains a challenge, however, to combine drought manipulation and isotopic labeling with concurrent measurements of NSCs and hydraulics for multiple plant species in a tropical forest.

To address this challenge, we conducted a unique rainfall exclusion and ^13^CO_2_ pulse-labeling experiment (the B2WALD campaign) in the Biosphere 2 Tropical Rainforest, AZ, USA (Werner *et al.*, 2021). Water supply was withheld from the rainforest for ~9.5 weeks to generate a rapid decline in soil water availability. We applied an ecosystem ^13^CO_2_ pulse-labeling prior to withholding water (pre-drought) and once again during the drought treatment to track the fate of newly assimilated photosynthates in three canopy and five understorey species. Such an ecosystem-scale labeling and manipulation experiment allowed us to simultaneously label a number of co-occurring canopy and understorey plants, and compare their NSC dynamics in response to drought while controlling for other environmental factors such as air temperature and soil properties. We collected leaves, phloem at the stem bases, and roots, and analyzed NSCs and ^13^C in soluble carbon over the course of the experiment. Furthermore, we measured midday leaf water potential, stomatal conductance and transpiration, and xylem sap flow as indicators of plant water use status. We expected that tropical forests under drought conditions would (i) mobilize carbohydrate storage to buffer reduced photosynthetic supply; and (ii) slow down export of carbohydrates from leaves and transport of carbohydrates below ground. Importantly, we hypothesized that (iii) the extent to which drought reduces carbohydrate storage and slows export and transport of carbohydrates would vary depending on the water use strategy and canopy position.

## Materials and methods

### Experimental design

The Biosphere 2 Tropical Rainforest ecosystem allows manipulation of environmental conditions such as rainfall, groundwater, temperature, and humidity beyond ranges typical of the variability that exists within Amazon forests (Smith *et al.*, 2020; Werner *et al.*, 2021). The ecosystem is enclosed with glass panels held together with mylar sheets that transmit ~60% of solar radiation. The light was measured with photosynthetically active radiation (PAR) sensors (Apogee SQ-110) every 15 min, and the period when the PAR is greater than zero at the top of the Biosphere 2 rainforest was considered daytime. During the experimental period, the ecosystem received a mean photosynthetic photon flux density of ~788 µmol m^–2^ s^–1^ during the day and the air temperature was between 21 °C and 37 °C (day) and 20 °C and 27 °C (night). It should be noted that some features of this system differ from natural environmental conditions, in particular the relatively low light intensities and the sandy soil. Soil water potential (WP) was measured at four soil pits located across the ecosystem. Shallow soil WP was averaged from WP measurements at soil depths of 5 cm and 10 cm. Deep soil WP was calculated based on soil moisture measured at the bottom of the four soil pits spanning from the soil surface to the concrete subsurface (depths: 180, 200, 290, and 310 cm) and the water retention curves (Werner *et al.*, 2021). Ecosystem fluxes were continuously measured as in Werner *et al.* (2021).

Drought manipulation was achieved by withholding rainfall from 8 October 2019 to 2 December 2019 (Supplementary Fig. S1). Starting on 1 November 2019, drought was exacerbated by actively reducing relative humidity through condensation, while also heating dry air to maintain temperatures and draining the water table in an isolated drainage basin. A unique ecosystem stable isotope pulse-labeling was conducted during pre-drought (5 October) and drought (23 November). Labeling was achieved by adding 99 atom % ^13^CO_2_ (pre-drought, 150 liters; drought, 300 liters) to the atmosphere at two ground-based locations in the morning, reaching a maximum δ^13^C value in atmospheric CO_2_ of 1183 ± 4‰ and 2541 ± 222‰ (2 min averages of 1 Hz measurements and their SDs) at the 20 m atmospheric inlet under pre-drought and drought conditions, respectively. The increased intensity and duration of ^13^CO_2_ labeling under drought were designed to ensure a similar uptake of ^13^CO_2_ label to that in pre-drought, despite significant reductions in assimilation rates during drought.

### Species selection, sample collection, and processing

The Biosphere 2 tropical forests had a leaf area index of ~4–5, similar to natural tropical forests. We selected eight plant species (three or four plants per species) for NSC analysis and isotopic tracing. These species represent a significant proportion of ecosystem biomass, including three canopy tree species: *Clitoria fairchildiana* (21.5 ± 3.1 m tall; emergent layer), *Phytolacca dioica* (10.8 ± 3.6 m), and *Pachira aquatica* (9.7 ± 5.5 m), and five understorey plant species (<5 m): *Piper auritum* (<5 m), *Hibiscus rosa sinensis* (<5 m), *Calathea* sp. (<2 m), *Syngonium* sp. (<1 m), and *Dieffenbachia* sp. (<2 m).

All leaves collected during the pre-drought and drought period were mature at the time of sampling. For most species, we observed very little new leaf formation during the 8 weeks of drought between the first and second labeling periods. One exception is *P. auritum*, where new leaves were clearly developing during drought, but we tried to avoid sampling these new leaves. Leaf sampling was conducted within a 1 h time frame in the late morning, with the assistance of climbers ascending the enclosure structure to access the upper canopy. Leaves were collected before each ^13^CO_2_ pulse-labeling event and 0, 1, 3, and 5 d after the labeling. For phloem and roots, sampling frequency was reduced to minimize disturbance to the plants and soil. Bark samples from the canopy trees were collected prior to labeling, 3–4 d after labeling, and 8–16 d after labeling. Bark samples were taken with a puncher (Ø=8 mm) at the stem bases of the canopy trees. The inner bark was carefully separated from the outer dead bark and xylem wood, and defined as phloem in this study. Leaf and inner bark samples were placed into plastic tubes and immediately frozen in liquid nitrogen or dry ice to stop metabolic activity. Roots were collected prior to each labeling and 3–4 d after the labeling. Lateral roots (>2 mm diameter) were carefully dug out from the upper 20 cm of soil with a spade. To ensure root identification to the specific tree, all sampled roots were traced back to the primary or secondary root. Root samples were washed free of soil within 30 min after sampling. Note that root samples are missing for the canopy species under drought conditions due to sample loss during the shipping process. All samples were stored in 50 ml plastic tubes under –20 °C until freeze-drying.

### Soluble sugar and starch analysis

All samples were ground to fine powder with a ball mill and analyzed for soluble sugars (glucose, sucrose, and fructose) and starch using the standard protocol of Landhäusser *et al.* (2018). Briefly, soluble sugars were extracted from freeze-dried powdered samples in 85% ethanol, and were analyzed by HPLC coupled to pulsed amperometric detection (HPLC-PAD). The remaining pellets after the sugar extraction were oven-dried and digested with α-amylase and amyloglucosidase (Sigma-Aldrich), and analyzed using HPLC-PAD to determine concentrations of hydrolyzed glucose. Total NSCs were calculated as the sum of soluble sugars and starch. All concentration data (including the ratio of soluble sugars to total NSCs) are reported in Supplementary Figs S2–S4.

### Isotopic analysis

The δ^13^C values of the compounds dissolved in 85% ethanol (hereafter referred to as soluble carbon) were analyzed as previously described in Huang *et al.* (2021). Briefly, an aliquot of ethanol extract was pipetted into a tin cup and dried at 40 °C, and the procedure was repeated to achieve 0.1 mg dry samples. Samples were analyzed with an elemental anlayzer coupled to an isotope-ratio mass spectrometer. All δ^13^C data are reported in Supplementary Figs S5–S9. We also analyzed δ^13^C of bulk samples (i.e. freeze-dried fine powder), as an indicator of net assimilation of the ^13^C-label. Acetanilide (–30.06‰) and caffeine (–40.46‰) were measured as calibration and quality control, respectively (Werner and Brand, 2001).

For leaves, we calculated the MRT of the ^13^C-label in soluble carbon. The interval between the first and second labeling events was 7 weeks, which was much longer than the MRT (usually <3 d) of the ^13^C-label in the leaf soluble carbon or bulk reported in this study and elsewhere (Ruehr *et al.*, 2009; Streit *et al.*, 2013; Dannoura *et al.*, 2018; Hesse *et al.*, 2018; Duangngam *et al.*, 2020). Thus, the residual ^13^C from the first pulse label was negligible for the second pulse label. This is also corroborated by similar δ^13^C values before the two pulse labels for three canopy species and two understorey species (*P*>0.1; Supplementary Figs S5, S6). However, the δ^13^C values before the second pulse label were higher than those before the first pulse label in *Calathea* sp. (*P*=0.04), *Dieffenbachia* sp. (*P*<0.01), and *P. auritum* (*P*=0.07), most probably due to reduced carbon discrimination during drought. Nonetheless, this difference was relatively small compared with the δ^13^C peak after the pulse label. In addition, during the 5 d of the labeling and sampling period under drought, there were no significant changes in carbon discrimination as stomatal conductance remained low and constant (Supplementary Fig. S10). To minimize effects from carbon isotopic discrimination under drought, we normalized ^13^C values (atom % ^13^C) as excess ^13^C relative to pre-labeling ^13^C values for each labeling campaign.

For stem phloem and roots, however, we did not calculate the MRT of the ^13^C-label due to limited sampling time points. In addition, the enrichment of the ^13^C-label was also much lower in the stem phloem and roots than in the leaves in all species, most probably as a result of continuous mixing of ^13^C-labeled photosynthates with older stored carbohydrates along the transport pathway (Furze *et al.*, 2018b; Tixier *et al.*, 2018). Thus, we use the appearance of the excess ^13^C in stem phloem and coarse root as an indicator of transport times of recent photosynthates. It should be noted that we extracted soluble carbon from phloem (i.e. inner bark) and root samples that contain not only recent photosynthates but also older stored soluble compounds. However, the initial appearance of the label (i.e. excess ^13^C relative to pre-labeling values) and its delay under drought can still be detected in *C. fairchildiana* and *P. dioica* by our approach.

We converted the δ^13^C value to ^13^C atom fraction in %, using the equation:


atom % C13=1001(δ13C1000 + 1)×0.0111802+1      (1)

where 0.0111802 is the isotope ratio of VPDB.

The excess ^13^C (i.e. attributed to the ^13^C-label) values were calculated by subtracting the pre-labeling ^13^C values (^13^C_pre-label_) from the ^13^C values after pulse-labeling (^13^C_labeled_):


Excess C13=C13labeled−C13pre−label (2)

We fitted an exponential decay function to the excess ^13^C values of leaf soluble carbon within each species, using the nls function in R:


$$\begin{array}{l} N\left(t\right)=N_{0}\times e^{-\lambda t} \end{array}$$ (3)

where *N*(*t*) is the measured excess ^13^C value at the time (*t*) after labeling, *N*_0_ is the maximal excess ^13^C signal that occurred immediately after labeling (i.e. day 0), and λ is the decay constant. The goodness of the exponential fit was assessed by Pearson’s correlations between measured and predicted excess ^13^C values (Supplementary Table S1). The MRT of the ^13^C-label in the leaf soluble carbon pool was then calculated as:


$$\begin{array}{l} MRT=\frac{1}{\lambda } \end{array}$$ (4)

### Hydraulic traits

Midday leaf water potential (MPa) was measured on sunlit leaves from five of the eight plant species (see Table 1). Measurements were carried out using a Scholander type pressure chamber between 10.00 h and 11.00 h, because previous studies from this experimental tropical forest showed that plant photosynthesis decreases as temperature increases after 11.00 h (Rascher *et al.*, 2004; Werner *et al.*, 2021). All leaves were measured prior to drought (25 September), and ~7–8 weeks after the start of the drought treatment (24 November and 1 December). Pre-drought leaf water potential of one *P. aquatica* tree was not available and was replaced by data (–0.35 MPa) recorded 2 weeks after the start of the drought treatment. This is justified given that leaf water potential in *P. aquatica* species remained relatively constant throughout the experiment. Pre-drought leaf water potential of *P. auritum* was not measured and was replaced by data (–0.33 ± 0.17 MPa) recorded 3 weeks after rewetting the system. We measured leaf water fluxes of these five species using custom-built flow-through leaf chambers (Kübert *et al.*, 2023).

**Table 1.** Plant water use strategy characterized by hydraulics

Species	Position	Drought Ψ_leaf_ (MPa)	Drought ∆Ψ_leaf_ (MPa)	Wood density (g cm^–3^)	% deviation in sap flow	% deviation in Tr	% deviation in Gs
*P. aquatica*	Subcanopy	−0.56 ± 0.16	−0.23 ± 0.16	0.32 ± 0.04	−31.8 ± 7.7	−88.0 ± 2.2	−97.3 ± 1.3
*P. auritum*	Understorey	−1.06 ± 0.06	**–**	**–**	**–**	−59.0 ± 6.4	−93.8 ± 2.2
*P. dioica*	Subcanopy	−1.03 ± 0.18	−0.57 ± 0.13	0.15 ± 0.02	**–**	−51.0 ± 8	−55.5 ± 11.2
*H. rosa sinensis*	Understorey	−1.76 ± 0.39	−1.39 ± 0.37	0.42	−43.2 ± 26.3	−64.5 ± 14	−76.8 ± 10.9
*C. fairchildiana*	Canopy	−1.39 ± 0.07	−0.92 ± 0.03	0.54 ± 0.01	−86.0 ± 2.5	−52.1 ± 6.7	−58.8 ± 10.0

Values are means (±SE) of three or four plants per species. Ψ_leaf_, midday leaf water potential; ∆Ψ_leaf_, absolute changes in Ψ_leaf_ relative to pre-drought values; % deviation, percentage deviations from pre-drought values; Tr, transpiration; Gs, stomatal conductance. See Supplementary Table S2 for absolute values of sap flow, Tr, and Gs.

Xylem sap flow (l d^–1^) was measured on the canopy species. Sap flow velocities were measured with heat-pulse velocity sap flow sensors installed 110–130 cm above ground. Baselining was conducted based on the daily mean minimum plateau, mostly occurring in the hours before dawn. The sap flow was calculated based on sap flux density (cm h^–1^) for both the outer and inner measurement location (0.5–1.5 cm and 1.5–2.5 cm into the tree) and assuming a continued radial decrease of velocities away from the bark and radial distribution of conductive tissues (Werner *et al.*, 2021). Wood density was measured on intact wood cores taken from the trees with a 5.15 mm or 0.2 inch increment borer (Haglof, Madison, MI, USA) according to the standard methods described in Pérez-Harguindeguy *et al.* (2016).

### Statistical analysis

NSC concentration data for each plant and organ type were averaged across the sampling days during the labeling campaign under pre-drought and peak drought conditions before statistical testing. Data normality and homogeneity were tested with the Shapiro–Wilk test and the Levene test, respectively. Data were log-transformed when assumption of normality or homogeneity was violated. We applied paired *t*-test for the leaf and phloem NSC concentration data of the three canopy species and *H. rosa sinensis* where samples were repetitively sampled on the same tree, and unpaired *t*-test for the other data due to random selections of plants per species or different sample sizes. Across species, differences between pre-drought and drought were tested using two-way ANOVA, with drought and species as independent variables and species means as dependent variables. We calculated percentage deviations within and across species using the difference of the means between drought and pre-drought divided by those pre-drought values (Adams *et al.*, 2017).

For hydraulic data, leaf water potential from each tree was averaged over the two sampling dates at peak drought, and daily sums of sap flow of each tree were averaged over 1 week before the drought treatment started and over the last week of the drought treatment. Because *C. fairchildiana* and *P. aquatica* had large within-species variability in sap flow (the coefficient of variation is 0.97 and 1.58 under pre-drought conditions, respectively), percentage deviation from pre-drought in sap flow and leaf water potential were calculated for individual trees and then averaged within the species.

For isotopic data, the amount of the ^13^C-label in leaves may vary depending on the leaf position in the canopy, stomatal conductance, and the amount of ^13^CO_2_ added to the ecosystem during pulse-labeling. Therefore, for each sampling day, we averaged ^13^C values of leaf soluble carbon within each species, and calculated and normalized excess ^13^C values as a percentage of the maximal excess ^13^C values in the leaves (immediately after labeling, denoted as day 0). The coefficient of variation was calculated as the measure of spread of the excess ^13^C data of leaf soluble carbon at day 0. The coefficient of variation was calculated for pre-labeling ^13^C data of phloem/root soluble carbon (day –1). Standard errors were propagated according to error propagation rules. Across species, we analyzed the effects of interactions of drought and species (independent variables) on soluble sugars, starch, NSCs, and excess ^13^C (dependent variables) using two-way ANOVA (the aov function in R) (Supplementary Tables S3, S4). All statistical analyses were conducted in R (version 4.1.1, R Development Core Team, 2016).

## Results

**Fig. 1.**
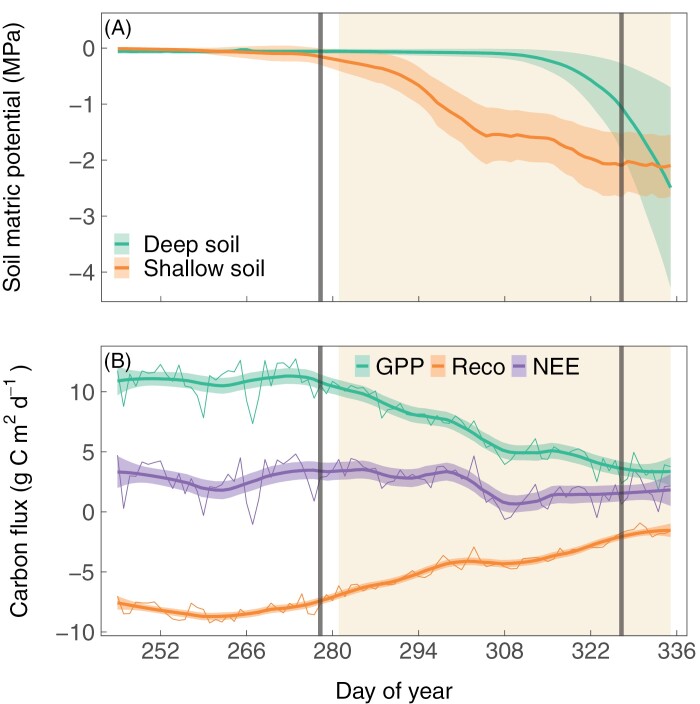
Changes in soil matric potential and ecosystem carbon fluxes over the course of the experiment. Daily means of (A) soil matric potential in the shallow (0–10 cm) and deep soil (2–3 m), and (B) ecosystem carbon fluxes include gross primary productivity (GPP), ecosystem respiration (Reco), and net ecosystem exchange (NEE). Background shadings indicate the drought days. Gray lines indicate the pulse-labeling events. Thick lines are smoothing splines with 95% confidence intervals. Modified from Werner *et al.* (2021). Reprinted with permission from AAAS.

At the ecosystem scale, we assessed how gross primary productivity (GPP), ecosystem respiration (Reco), and net ecosystem exchange (NEE) changed over the course of the experiment (see Werner *et al.*, 2021 for more results and methodologies). Briefly, as soil matric potential declined in the shallow soil (0–10 cm) and deep soil (2–3 m) relative to pre-drought conditions (Fig. 1A), both GPP and Reco (absolute value) declined (Fig. 1B). As a result, there was a slight decline in NEE.

We assessed the water use status of five plant species by measuring leaf water potential, stomatal conductance, woody density, and sap flow (Table 1): (i) *P. aquatica* and *P. dioica* had relatively higher and constant leaf water potential during drought than *H. rosa sinensis* and *C. fairchildiana*; (ii) *P. aquatica* and *P. auritum* had relatively higher stomatal sensitivity to drought than *P. dioica*, *H. rosa sinensis*, and *C. fairchildiana*; and (iii) *P. aquatica* had relatively lower wood density and smaller reductions in sap flow during drought than *C. fairchildiana*. Thus, *P. aquatica* and *P. auritum* had a more conservative water use strategy than *H. rosa sinensis* and *C. fairchildiana*, while *P. dioica* was intermediate in terms of water use during drought.

**Fig. 2.**
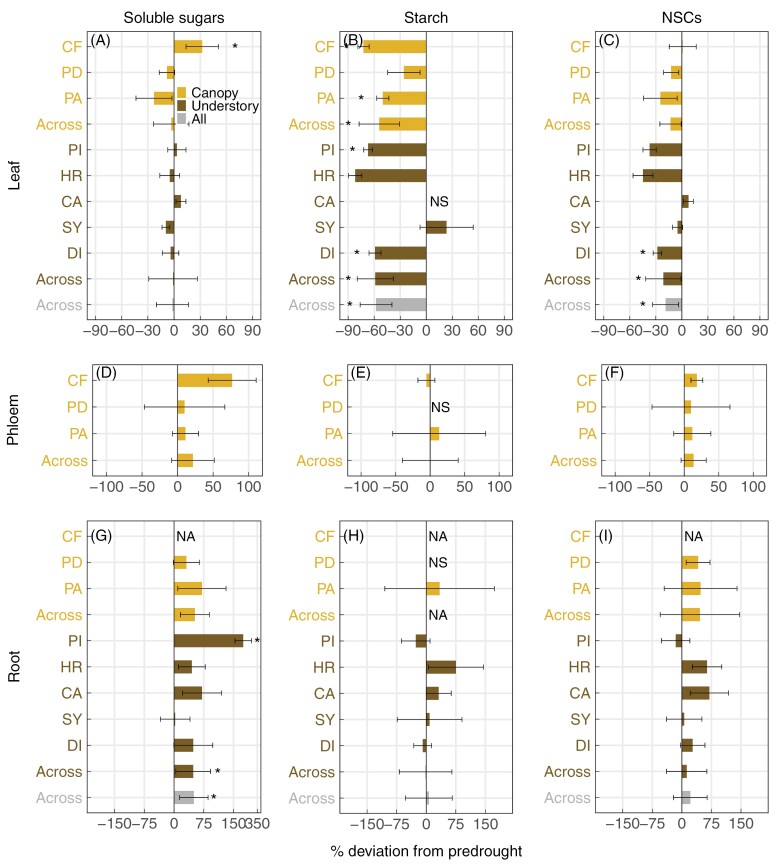
Impacts of drought on non-structural carbohydrates (NSCs). Relative changes in concentrations of soluble sugars, starch, and total NSCs (sugars+starch) in the leaves (A–C), phloem (D–F), and roots (G–I) under drought, expressed as percentage deviations from pre-drought values (*n*=3 or 4 plants per species). Positive values represent an increase under drought and negative values represent a decrease. Error bars represent SEs. Data are shown for within and across the three canopy species (light brown), namely *Clitoria fairchildiana* (CF; less conservative), *Phytolacca dioica* (PD; intermediate), and *Pachira aquatica* (PA; more conservative), within and across the five understorey species (dark brown), namely *Piper auritum* (PI), *Hibiscus rosa sinensis* (HR), *Calathea* sp. (CA), *Syngonium* sp. (SY), and *Dieffenbachia* sp. (DI), as well as across all species (gray). Data are not available (NA) for CF root. Percentage deviations were not computed (NS) for starch in CA leaves, PD phloem and root due to very low pre-drought concentrations (<0.5%; Supplementary Figs S2, S3). Significant within-species (Student’s *t*-test) and cross-species (two-way ANOVA; Type I sum of squares) differences between pre-drought and drought were calculated based on the raw concentrations and are indicated by an asterisk (*P*<0.05).

We examined changes in NSCs across organs to investigate how tropical plants use NSCs in response to drought (Fig 2; Supplementary Table S3). Among canopy species, leaf sugar concentrations increased in *C. fairchildiana* and remained low (2%) and constant in *P. dioica*; however, drought led to a modest decline in the levels of soluble sugars in the leaves of *P. aquatica*. (Fig 2A; Supplementary Fig. S2; Supplementary Table S5). Across five understorey species, leaf sugar concentrations remained relatively constant during drought (Fig 2A; Supplementary Table S3), and were relatively low in the two understorey species *P*. *auritum* (1%) and *H. rosa sinensis* (2%) throughout the experiment (Supplementary Fig. S3). Unlike leaf sugars, leaf starch was strongly depleted relative to the pre-drought conditions in five species (Fig 2B), including the canopy species *C. fairchildiana* and *P. aquatica* (Supplementary Fig. S2; Supplementary Table S5), and the understorey species *P*. *auritum*, *Dieffenbachia* sp., and *H. rosa sinensis* (Supplementary Fig. S3; Supplementary Table S6). Leaf starch concentrations remain low (<0.7%) in the canopy species *P. aquatica* (Supplementary Fig. S2) and the understorey species *Calathea* and *Syngonium* (Supplementary Fig. S3). This led to, on average, a declining trend in total NSCs across species (Fig. 2C; Supplementary Table S3), and an increase in the proportion of soluble sugars in total NSCs in many species (Supplementary Fig. S4).

In stem phloem and roots, soluble sugars and starch showed large variations within species (Fig. 2). Drought appeared to increase concentrations of soluble sugars (Fig. 2D, G), especially in the phloem of the canopy species *C. fairchildiana* and in the roots of the understorey *P*. *auritum*. Across species, concentrations of root soluble sugars significantly increased under drought (Fig. 2G), while starch concentrations remained relatively constant (Fig. 2H). Starch was absent in the stem phloem of the canopy species *P. dioica* and the roots of understorey *Calathea* sp. (<0.1%; Supplementary Figs S2, S3).

**Fig. 3.**
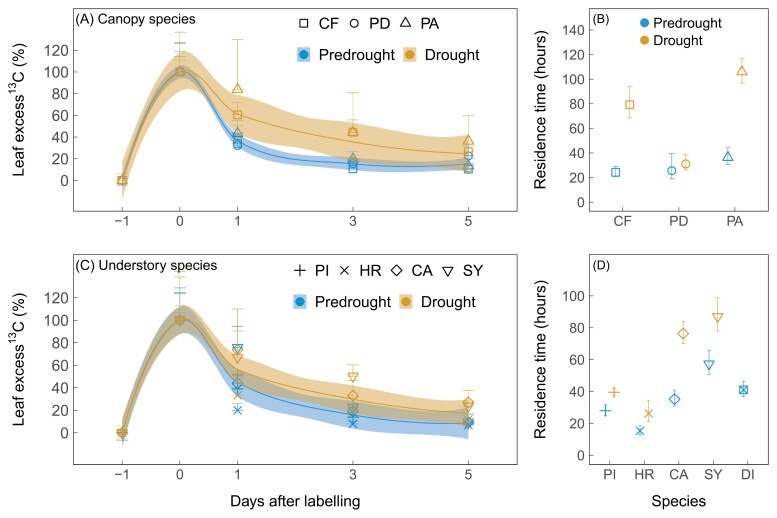
Impacts of drought on the temporal dynamics of recent photosynthates in leaves. Temporal changes and the mean residence time of the ^13^C label (the excess ^13^C relative to the pre-labeling ^13^C values in atom %) in leaf soluble carbon under pre-drought and drought conditions (*n*=3 or 4 plants per species). Data are shown for the three canopy species, namely *Clitoria fairchildiana* (CF), *Phytolacca dioica* (PD), and *Pachira aquatica* (PA), and five understorey species, namely *Piper auritum* (PI), *Hibiscus rosa sinensis* (HR), *Calathea* sp. (CA), *Syngonium* sp. (SY), and *Dieffenbachia* sp. (DI). In the left panels, data are expressed as a percentage of the maximum excess ^13^C signals that occurred immediately after labeling (day 0), and error bars represent the coefficient of variation for day 0 and propagated SEs for other days (–1, 1, 3, 5). Blue (pre-drought) and yellow (drought) shaded areas represent 95% confidence intervals of the regression lines across species. In the right panels, isotopic data were averaged within species on each sampling day to fit an exponential decay function to calculate the MRT; error bars represent SEs from estimating the MRT. To improve clarity, the temporal dynamics of the excess ^13^C in DI leaves under drought were not shown because it peaked at day 1 rather than at day 0 (Supplementary Fig. S6). This also prevents the fitting of a non-linear least squares model to the data for calculations of MRT.

We examined the temporal dynamics of the ^13^C label in the leaf soluble carbon pool to investigate how drought affects export of recent photosynthates (Fig. 3; Supplementary Figs S5, S6; Supplementary Table S4). We found that excess ^13^C in the leaf soluble carbon pool peaked immediately after labeling and declined exponentially over time across canopy and understorey species (Fig. 3A, C), except for the understorey species *Dieffenbachia* sp. where excess ^13^C peaked at day 1 under drought (Supplementary Fig. S6). When accounting for the addition of twice as much ^13^CO_2_ label to the atmosphere under drought, there was a significant decrease in the leaf uptake of ^13^CO_2_-label at day 0 (Supplementary Table S4), with the most pronounced decrease observed in *P. aquatica* and *Dieffenbachia* sp. (Supplementary Figs S5, S6). Under pre-drought conditions, the MRT of the ^13^C-label in leaf soluble carbon ranged between 20 h and 70 h (Fig. 3B, D). Drought generally increased the MRT of the ^13^C-label in leaf soluble carbon across canopy and understorey plant species (Fig. 3B, D).

**Fig. 4.**
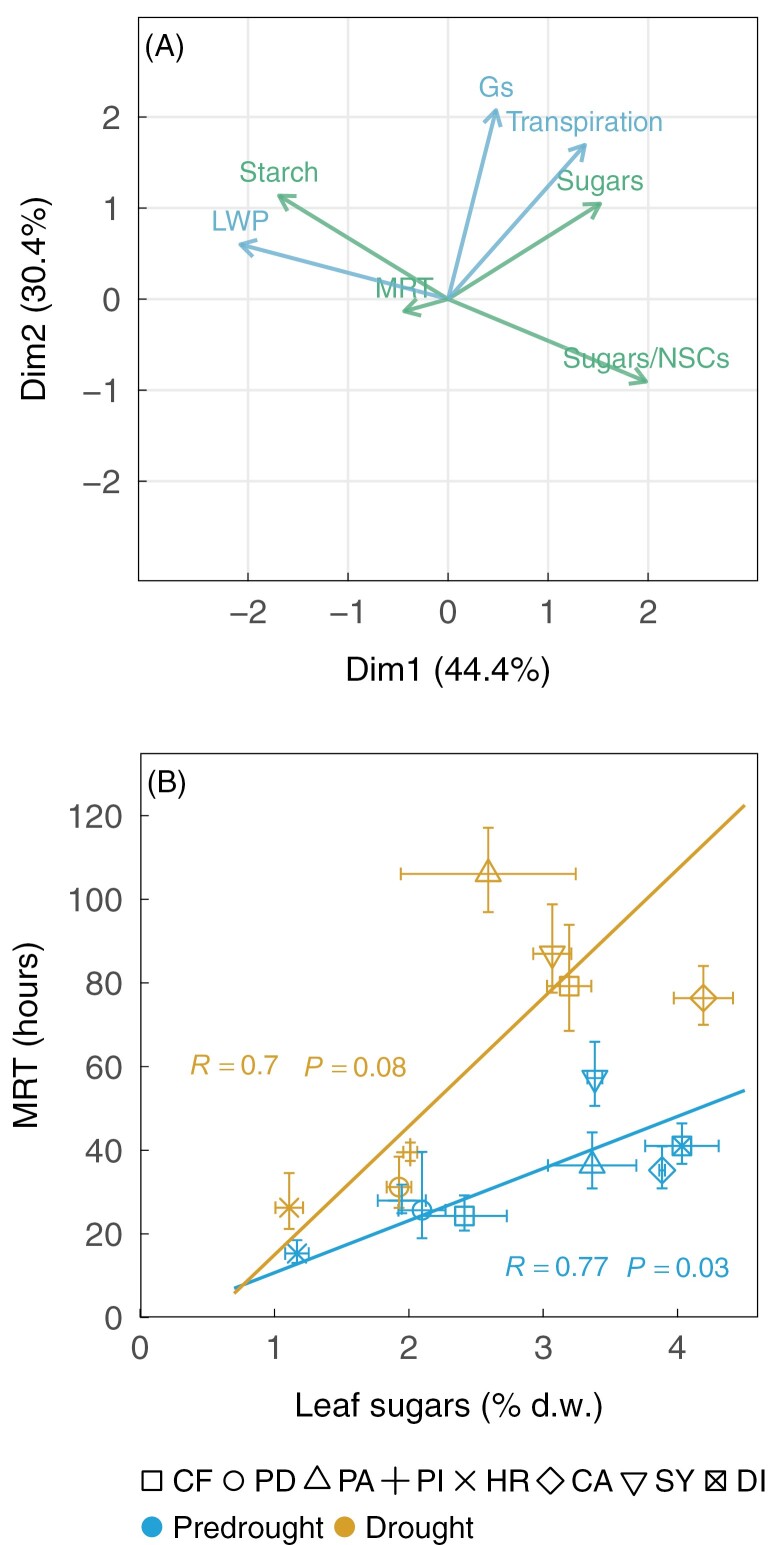
Relationships between leaf hydraulics, non-structural carbohydrates (NSCs), and the mean residence time (MRT) of recent photosynthates. A principal component analysis (A) summarizing relationships between drought-induced changes (i.e. percentage deviation from pre-drought) in hydraulic traits (blue) represented by leaf water potential (LWP), leaf transpiration and stomatal conductance (Gs), and carbohydrate status represented (green) by leaf soluble sugars, starch, the ratio of soluble sugars to NSCs (sugars/NSCs), and the MRT of recent photosynthates. Data are based on the five species listed in Table 1. The relationships (B) between concentrations of leaf soluble sugars and the absolute MRT (hours) of the ^13^C label in leaf soluble carbon across the three canopy species (*Clitoria fairchildiana*, CF; *Phytolacca dioica*, PD; *Pachira aquatica*, PA) and five understorey species (*Piper auritum*, PI; *Hibiscus rosa sinensis*, HR; *Calathea* sp., CA; *Syngonium* sp., SY; *Dieffenbachia* sp., DI) under pre-drought (blue, solid line) and drought (yellow, dashed line) conditions (*n*=3 or 4 plants per species).

We performed principal component analysis (PCA) to examine relationships between drought-induced changes in NSCs and hydraulic traits (Fig. 4A). Changes in leaf starch and leaf sugars/NSCs were associated with changes in leaf water potential (Fig. 4A). Moreover, changes in leaf soluble sugars appeared to be associated with those in leaf transpiration and to a lesser extent stomatal conductance during drought. However, changes in the MRT of recent photosynthates were poorly represented on the principal component (Fig. 4A). We also performed correlations between the absolute MRT of recent photosynthates and leaf sugar concentrations across species to test whether the MRT of recent photosynthates depends on the size of the soluble sugar pool and whether drought affected that relationship (Fig. 4B). Under pre-drought conditions, species with a higher MRT of the ^13^C-label also had significantly higher concentrations of leaf soluble sugars (*R*=0.77, *P*<0.05). Drought affected the relationships by increasing the MRT, especially in species with relatively high concentrations of leaf soluble sugars (Fig. 4B).

**Fig. 5.**
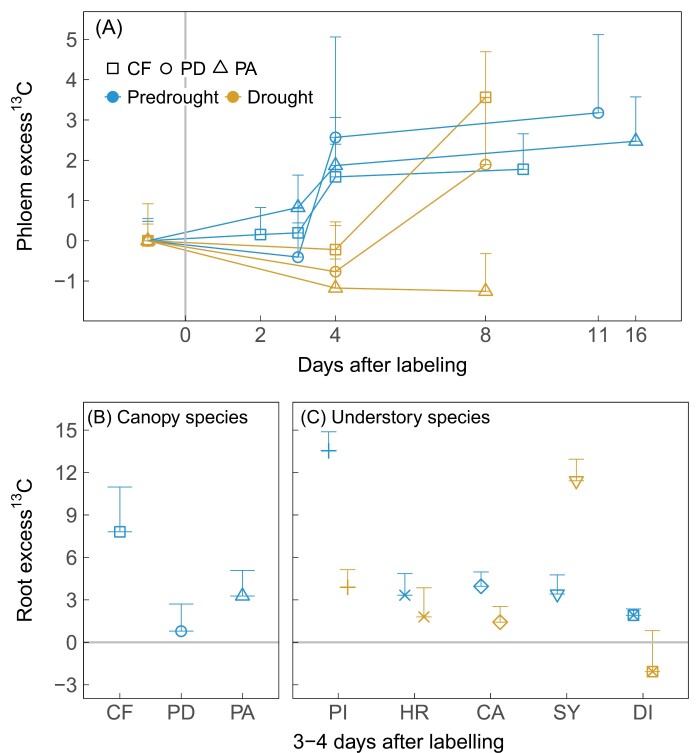
Impacts of drought on the transport of recent photosynthates. Temporal changes of the ^13^C label (the excess ^13^C relative to the pre-labeling ^13^C values in atom %) in soluble carbon in the stem phloem (A) and roots (B, C) under pre-drought and drought conditions (*n*=3 or 4 plants per species). Stem phloem data are shown for the three canopy species, namely *Clitoria fairchildiana* (CF), *Phytolacca dioica* (PD), and *Pachira aquatica* (PA). Root data are shown for the canopy species, as well as understorey species *Piper auritum* (PI), *Hibiscus rosa sinensis* (HR), *Calathea* sp. (CA), *Syngonium* sp. (SY), and *Dieffenbachia* sp. (DI) within 4 d after labeling. Note that root ^13^C data are missing for the canopy species under drought conditions due to sample loss during the shipping process. For clarity, all values were multiplied by 1000. Error bars represent the coefficient of variation for pre-labeling (day –1) and propagated SEs after labeling.

We tracked the ^13^C-label in the soluble carbon pool from the leaves to the phloem at the stem bases and roots to investigate how drought affects transport of recent photosynthates (Fig. 5; Supplementary Figs S7–S9). Across all canopy species, phloem at the stem bases showed a ^13^C enrichment of soluble carbon 3–4 d after the pre-drought labeling (Fig. 5A). We also found ^13^C enrichment of root soluble carbon in two canopy species, *C. fairchildiana* and *P. aquatica*, 4 d after labeling (Fig. 5B). Under drought, however, no ^13^C enrichment was observed in the phloem 4 d after labeling, and for *P. aquatica* not even at 8 d after labeling (Fig. 5A; Supplementary Table S4). For understorey species, we were not able to determine when ^13^C-labeled compounds arrived at the roots due to limited root sample size. However, ^13^C enrichment in root soluble carbon was lower under drought than under pre-drought in all understorey species except for *Syngonium* sp. (Fig. 5C).

## Discussion

We hypothesized that tropical plants would mobilize stored NSCs, in particular starch, to buffer reductions in carbon uptake during drought. This was observed in leaves, where starch levels strongly declined under drought (Fig. 2B). However, our results contrast with the results of Pivovaroff *et al.* (2021) and Zhang *et al.* (2020), who found homeostatic levels of leaf starch in (sub)tropical trees under experimental drought. These seemingly contradicting results are likely to be due to different drought intensities (McDowell *et al.*, 2011, 2022), where photosynthesis and starch storage remained relatively constant under long-term relatively moderate drought treatment (~40% and 70% rainfall exclusion) in Pivovaroff *et al.* (2021) and Zhang *et al.* (2020), respectively, but were reduced under short-term severe drought treatment (100% rainfall exclusion) in our study. It is worth noting that these tropical plants reduced carbon assimilation and depleted leaf starch in response to shallow soil drying, while a large proportion of water was still available in the deep soil layer. This indicates that reduced root water uptake from the shallow soil during drought cannot be compensated by water uptake from the deep soil, consistent with results in a beech forest (Gessler *et al.*, 2021). Our results further showed that tropical plants tend to convert leaf starch to soluble sugars, probably to meet high demand for osmotic regulation in response to reductions in leaf water potential under drought (Hajek *et al.*, 2022; Blumstein *et al.*, 2023). This is supported by strong relationships between increased sugars/NSCs (the proportion of soluble sugars in NSCs) and decreased leaf water potential under drought (Fig. 4), consistent with results of recent studies conducted in tropical forests (Signori-Müller *et al.*, 2021).

Despite reductions in leaf starch across species under drought, changes in leaf soluble sugars appear to vary depending on the canopy position and plant water status. *Clitoria fairchildiana* is the only species that increased concentrations of leaf soluble sugar contents under drought (Fig. 2; Supplementary Fig. S2; Supplementary Table S5). As the dominant canopy-forming trees species, *C. fairchildiana* is exposed to more sunlight, warmer temperature, and lower humidity, leading to higher demand for evapotranspiration. Still, *C. fairchildiana* maintained stomata partially open under drought (Table 1), leading to deteriorated water status (as indicated by significant reductions in leaf water potential) while allowing continued photosynthesis and production of carbohydrates. For the subcanopy and understorey species, however, changes in leaf soluble sugars were likely to be strongly affected by light limitation (Zhang *et al.*, 2020; Rowland *et al.*, 2021). In particular, the conservative subcanopy species, *P. aquatica*, showed a decrease in leaf soluble sugars and total NSCs under drought (Fig. 2; Supplementary Fig. S2; Supplementary Table S5), most probably due to the combination of low light and strict stomatal control.

For stem phloem and roots, however, starch levels showed large variations and remained high in most species under drought (Fig. 2). Substantial amounts of NSCs are stored in xylem sapwood and tap roots over a time scale of years to decades (Hoch *et al.*, 2003; Furze *et al.*, 2018a; Herrera-Ramírez *et al.*, 2021), and can be remobilized to phloem and coarse roots to buffer changes in the supply of recent photosynthates under drought (Muhr *et al.*, 2018; Peltier *et al.*, 2023). For example, it has been shown that NSCs in the coarse roots remained constant for 3–12 months or even several years after complete termination of the supply of recent photosynthates via stem girdling (Aubrey and Teskey, 2018; Rademacher *et al.*, 2021; Helm *et al.*, 2023). Hence, stem phloem and coarse roots were unlikely to be carbon limited after the 8 week drought. Instead, some species even increased concentrations of NSCs and soluble sugars in these organs, possibly due to reduced demands for growth and respiration (Fatichi *et al.*, 2014; Adams *et al.*, 2015; Oberleitner *et al.*, 2022) and increased demands for osmoregulation. This is especially true for the dominant canopy trees (*C. fairchildiana*), where xylem sap flow was strongly reduced under drought. Collectively, these results indicate that, despite reduced supply of new photosynthates (see below), stem phloem and coarse roots may depend on remobilization of older storage reserves for osmotic regulation under drought.

We hypothesized that drought would slow export of recently assimilated photosynthates from leaves. This is supported by an increase in the MRT of recently assimilated carbohydrates in leaf soluble carbon under drought (Fig. 3), which is in line with the observed increased MRT of stem ^13^C respiration (Werner *et al.*, 2021). Our results suggest that drought did not reduce incorporation of recent carbohydrates to other pools, such as leaf non-soluble carbon pools (Supplementary Fig. S11) and night-time respiration (Werner *et al.*, 2021). Therefore, reduced carbohydrate export from leaves is most likely to be responsible for the increased MRT under drought. Although drought impacts on carbohydrate export did not vary strongly with the water use or position of the species in the forest canopy, the mechanisms leading to slower carbohydrate export under drought are likely to be different for the canopy species with different water-use strategies (Sala *et al.*, 2010; Salmon *et al.*, 2019): *C. fairchildiana* with a water-spending strategy increased concentrations of leaf soluble sugars as osmolytes to ensure osmoregulation (Table 1), potentially rendering these osmolytes less accessible for export under drought. By contrast, *P. aquatica* with a conservative water use strategy strongly reduced photosynthesis, as indicated by lower incorporation of the ^13^C-label under drought (Supplementary Fig. S5), leading to a lower carbohydrate pool for export (Fig. 2).

The MRT of recent photosynthates appears to increase with the size of the soluble sugar pool, independent of canopy positions and water use strategies (Fig. 4; Supplementary Fig. S12). Interestingly, drought impacts on the MRT of recent photosynthates were found to be less pronounced for species with constant and lower levels of soluble sugars (<2%; Fig. 4). This level of soluble sugars has been observed in seedlings experiencing severe carbon limitation (Wiley *et al.*, 2017; Weber *et al.*, 2018), suggesting that tropical species with low levels of soluble sugars may preferentially maintain fast turnover of recent photosynthates to ensure basal respiration (Huang *et al.*, 2021) or production of volatiles (e.g. monoterpenes) (Huang *et al.*, 2018; Byron *et al.*, 2022). By contrast, tropical species with higher levels of older, stored sugars are less dependent on recent photosynthates, especially under drought conditions. These results suggest that hydraulic constraints on mobilization of recent photosynthates (Sevanto *et al.*, 2014) may also increase with the size of the soluble sugar pool.

We hypothesized that drought can slow down phloem transport in this tropical forest. Consistent with our hypothesis, the transport time of ^13^C-labeled photosynthates from leaves to stem bases increased under drought for all canopy species (Fig. 5A). This provides a mechanistic interpretation for slow/reduced utilization of recent photosynthates for stem and below-ground respiration under drought (Werner *et al.*, 2021). Interestingly, among the canopy species, the appearance of ^13^C-label was delayed in *C. fairchildiana*, and not observed 8 d after labeling in the more conservative species *P. aquatica* under drought (Fig. 5A). This suggests that the impacts of drought on transport of recent photosynthates are more negative in species with a more conservative water use strategy. We propose two inter-related mechanisms leading to slower/reduced transport of recently assimilated carbohydrates in co-occurring tropical trees under drought: (i) longer MRT (i.e. slower export) of recent photosynthates in the leaves across species (Fig. 2); and (ii) reduced assimilation of recent photosynthates after stomatal closure (Table 1; Supplementary Fig. S5), especially for the conservative canopy species *P. aquatica*. These processes, combined with the continuous mixing of ^13^C-labeled photosynthates with older stored carbohydrates along the transport pathway (Furze *et al.*, 2018; Tixier *et al.*, 2018), may explain why we did not observe ^13^C enrichment in the stem phloem in *P. aquatica*. For understorey species, drought reduced the amount of ^13^C enrichment in root soluble carbon at day 4, except for *Syngonium* (Fig. 5C), even when canopy leaf area (approximately <5% leaf loss under drought) and the uptake of ^13^CO_2_ remained relatively constant or increased (Supplementary Fig. S6). These combined results also indicate that drought slowed down transport of recent photosynthates to the roots in most understorey species.

We note that our experiment was based on comparisons over time relative to strong environmental forcings (here before and during drought). As such, our results can be influenced by changes in climate and phenology. However, the enclosed evergreen rainforest in Biosphere 2 has low year-to-year and seasonal variability relative to external ecosystems, making time comparisons more robust. In both the 2002 (Rascher *et al.*, 2004) and 2019 (Werner *et al.*, 2021) drought experiments at the Biosphere 2 rainforest, GPP decreased during drought and rapidly increased following the return of water to the system (Supplementary Fig. S13), demonstrating that seasonal changes in temperature and light were not the primary drivers of reduced ecosystem productivity during drought. These results give us confidence that the observed responses in allocation of ^13^C labels, NSCs, and hydraulics were mainly caused by the imposed drought.

### Implications

**Fig. 6.**
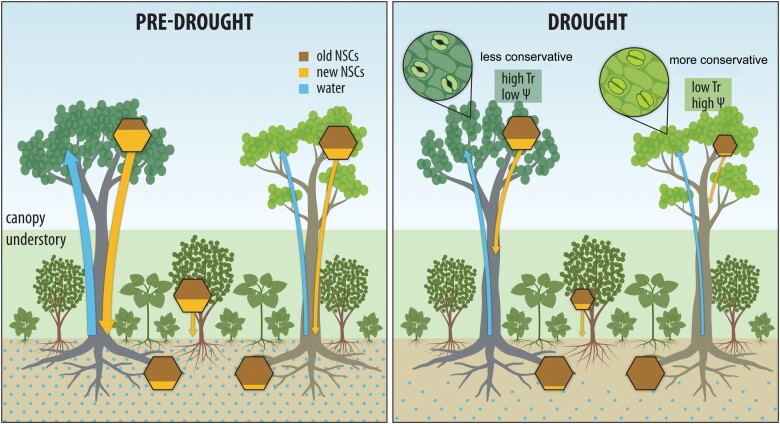
Schematic of the inter-related plant non-structural carbohydrate (NSC) dynamics and water use in the Biosphere 2 rainforest under drought. Changes in the concentration and flow of NSCs are indicated by the size of hexagons and arrows, respectively. Under drought, canopy trees (e.g. *P. aquatica*) with a more conservative water use strategy close stomata to avoid water loss via transpiration (low Tr) and maintain water status (Table 1; relatively high and constant leaf water potential and sap flow), while supply of newly assimilated NSCs (yellow fraction of the hexagons) is strongly reduced and mobilization of old leaf NSC storage (brown fraction) is required to support carbohydrate metabolism (respiration) (Werner *et al.*, 2021). By contrast, canopy trees with a less conservative water use strategy (e.g. *C. fairchildiana*) tend to maintain stomatal conductance and fuel soluble sugar pools with a continuous supply of newly assimilated NSCs, while experiencing deteriorated water status as evidenced by reduced water potential and sap flow under drought; this strategy leads to persistent structural damage (e.g. losses of leaves and hydraulic conductance), with significant impacts on ecosystem fluxes during drought and recovery (Werner *et al.*, 2021). Across understorey species, old leaf NSC storage is reduced as starch is depleted for metabolism, whereas soluble sugars are maintained for osmoregulation under drought. At the whole-plant level, drought slows/reduces leaf export of newly assimilated NSCs and phloem transport, especially for canopy trees with a conservative water use strategy. However, old NSC storage in below-ground woody organs is mobilized in order to buffer reductions in the supply of newly assimilated NSCs across species under drought.

Our results revealed mechanistic links between plant water use and NSC dynamics, with strong implications for understanding patterns of resistance and recovery as well as the dynamics of ecosystem fluxes in tropical forests during drought (Fig. 6). In the Biosphere 2 rainforest, mobilization of leaf storage reserves allows tropical plants to maintain water status and thus enhance resistance to drought (Werner *et al.*, 2021), especially for the canopy species with a conservative water use strategy (e.g. *P. aquatica*). However, for the canopy species (e.g. *C. fairchildiana*) with a less conservative water use strategy, NSC homoeostasis sustained by newly assimilated photosynthates is associated with substantial water loss and structural damage (losses of leaves) during drought, and incomplete recovery of canopy fluxes even after 2 months of drought release (Joseph *et al.*, 2020; Werner *et al.*, 2021; Hikino *et al.*, 2022). Under recurrent drought, this may cause structural damage to accumulate (Kannenberg *et al.*, 2019; Anderegg *et al.*, 2020; Oberleitner *et al.*, 2022), negatively impacting ecosystem primary production since *C. fairchildiana* is the largest contributor to total ecosystem fluxes of the Biosphere 2 rainforest (Werner *et al.*, 2021).

Slower/reduced export and transport of recent photosynthates under drought may lead to a shift in allocation of newly assimilated carbon to leaves rather than below ground, to preserve new photosynthates for leaf carbohydrate metabolism in species with low photosynthetic supply and for leaf osmotic regulation in species with deteriorated water status (Fig. 6). However, old storage reserves in stem and roots can play a crucial role in sustaining metabolism under drought. This is especially true for the conservative canopy trees, where supply of recent carbohydrates is strongly limited as a result of stomatal closure under drought (Fig. 6). Plant water use may thus determine the processes underlying how drought limits ecosystem carbon cycling (i.e. assimilation and allocation of recently assimilated carbohydrates) in tropical forests.

A more mechanistic understanding of tropical forest carbon cycling can be achieved via integrating the ^13^C and NSC concentration data with a range of physiological (e.g. photosynthesis), morphological (e.g. leaf area index and leaf mass per area), anatomical (e.g. phloem and sap wood area), and allometric traits (e.g. biomass ratios) in future ecosystem-scale labeling and manipulation experiments. Such experiments should also assess the partitioning of totally assimilated ^13^C labels to multiple sinks such as growth and respiration (Rog *et al.*, 2021; Hikino *et al.*, 2022), storage and defense (Huang *et al.*, 2019), and export as root exudation (Jiang *et al.*, 2023). Furthermore, our study highlights that vegetation models should include plant functional types based on the coupled NSC dynamics and water use strategies for reliable predictions of whole-tree carbon allocation patterns and ecosystem flux dynamics in tropical forests under drought.

## Supplementary data

The following supplementary data are available at *JXB* online.

Table S1. The coefficients and significance of the Pearson’s correlations between measured and predicted excess ^13^C values.

Table S2. The daily sum of sap flow, transpiration, and stomatal conductance under pre-drought and drought.

Table S3. Two-way ANOVA testing effects of drought and species and their interactions on leaf soluble sugars, starch, and NSCs.

Table S4. Two-way ANOVA testing effects of drought and species and their interactions on the amount of the ^13^C label.

Table S5. The coefficients and significance of the linear regressions of sugars, starch, and NSCs versus time for the three canopy species.

Table S6. The coefficients and significance of the linear regressions of sugars, starch, and NSCs versus time for the five understorey species.

Fig. S1. The drought treatment, labeling, and sampling timeline.

Fig. S2. Concentrations of soluble sugars, starch, and non-structural carbohydrates in the leaves and stem phloem in the three canopy tree species.

Fig. S3. Concentrations of soluble sugars, starch, and non-structural carbohydrates in the leaves and roots in the five understorey species.

Fig. S4. Relative changes in the ratio of soluble sugars to non-structural carbohydrates.

Fig. S5. The δ^13^C of leaf soluble carbon in the three canopy species before and after labeling.

Fig. S6. The δ^13^C of leaf soluble carbon in the five understorey species before and after labeling.

Fig. S7. The δ^13^C of phloem soluble carbon in the three canopy species before and after labeling.

Fig. S8. The δ^13^C of root soluble carbon in the three canopy species before and after labeling.

Fig. S9. The δ^13^C of root soluble carbon in the five understorey species before and after labeling.

Fig. S10. Changes in stomatal conductance during drought.

Fig. S11. The relationships between δ^13^C in the leaf soluble carbon and non-soluble carbon fractions under pre-drought and drought conditions.

Fig. S12. The relationships between midday leaf water potential and the absolute MRT of the ^13^C label in the leaf soluble carbon.

Fig. S13. Changes in gross primary productivity in two drought experiments at the Biosphere 2 rainforest.

erae030_suppl_Supplementary_Tables_S1-S6_Figures_S1-S13

## Data Availability

The ecosystem carbon flux and soil water potential data used in this manuscript are publicly available (https://doi.org/10.25422/azu.data.14632593). All NSC and isotope data associated with the manuscript are deposited in the TRY database (Dataset ID 845).
