# Supplementary material for: The mobilization and transport of newly fixed carbon are driven by plant water use in an experimental rainforest under drought
Source: J Exp Bot. 2024 Jan 25;75(8):2545–57. doi: 10.1093/jxb/erae030 (PMC11358253; doi:10.1093/jxb/erae030)
Supplement: erae030_suppl_Supplementary_Tables_S1-S6_Figures_S1-S13 [file erae030_suppl_supplementary_tables_s1-s6_figures_s1-s13.pdf]

**Supporting Information for**

# The mobilization and transport of newly-fixed carbon are driven by plant water-use in an experimental rainforest under drought

Jianbei Huang *, S. Nemiah Ladd, Johannes Ingrisch, Angelika Kübert, Laura K. Meredith, Joost van Haren, Ines Bamberger, L. Erik Daber, Kathrin Kühnhammer, Kinzie Bailey, Jia Hu, Jane Fudyma, Lingling Shi, Michaela A. Dippold, Kathiravan Meeran, Luke Miller, Michael J. O'Brien, Hui Yang, David Herrera-Ramírez, Henrik Hartmann, Susan Trumbore, Michael Bahn, Christiane Werner, and Marco M. Lehmann

*Jianbei Huang
Email: hjianbei@bgc-jena.mpg.de

**Table S1.** The coefficients and significance of the Pearson's correlations between measured and predicted excess $^{13}C$ values.

| Position | Species | Pre-drought | | | Drought | | |
|---|---|---|---|---|---|---|---|
| | | *R* | *P* | *df* | *R* | *P* | *df* |
| Canopy species | *Clitoria fairchildiana* | **0.99** | **< 0.01** | 2 | **0.97** | **< 0.05** | 2 |
| | *Phytolacca dioica* | **0.97** | **< 0.05** | 2 | **0.99** | **< 0.05** | 2 |
| | *Pachira aquatica* | **0.99** | **< 0.05** | 2 | **0.99** | **< 0.05** | 2 |
| Understory species | *Piper auritum* | **0.99** | 0.06 | 1 | **0.99** | **< 0.01** | 2 |
| | *Hibiscus rosa sinensis* | **0.99** | **< 0.01** | 2 | **0.99** | **< 0.05** | 2 |
| | *Calathea sp.* | **0.99** | **< 0.01** | 2 | **0.99** | **< 0.01** | 2 |
| | *Syngonium sp.* | **0.99** | **< 0.01** | 2 | **0.98** | **< 0.05** | 2 |
| | *Diefenbachia sp.* | **0.99** | **< 0.01** | 2 | NA | NA | NA |

**Table S2.** Absolute values of plant hydraulics under pre-drought and under drought.

| Species | Position | SF (Pre-drought) | SF (Drought) | Tr (Pre-drought) | Tr (Drought) | Gs (Pre-drought) | Gs (Drought) |
|---|---|---|---|---|---|---|---|
| *P. aquatica* | Subcanopy | 1.5 ± 1.4 | 1.0 ± 0.9 | 0.25 ± 0.09 | 0.03 ± 0.01 | 63.2 ± 20.6 | 1.2 ± 0.5 |
| *P. auritum* | Understory | - | - | 0.33 ± 0.02 | 0.13 ± 0.02 | 136.4 ± 31.4 | 6.7 ± 0.6 |
| *P. dioica* | Subcanopy | - | - | 0.71 ± 0.23 | 0.40 ± 0.19 | 30.8 ± 8.5 | 15.9 ± 7.6 |
| *H. rosa sinensis* | Understory | 1.8±0.7 | 0.7±0.2 | 0.24 ± 0.11 | 0.06 ± 0.02 | 18.9 ± 11.9 | 1.8 ± 0.7 |
| *C. fairchildiana* | Canopy | 22.1±10.7 | 3.7±2.4 | 0.40 ± 0.08 | 0.18 ± 0.04 | 27.8 ± 11.7 | 8.9 ± 3.0 |

Values are means (±SE) of 3 or 4 plants per species. SF, sap flow (L/day); Tr, transpiration (mmol $m^{-2}$ $s^{-1}$); Gs, stomatal conductance (mmol $m^{-2}$ $s^{-1}$).

**Table S3.** Two-way ANOVA testing effects of drought and species and their interactions on concentrations of soluble sugars, starch and total NSCs in leaves.

| Species | Pools | Species (*Df*) | Species (*F value*) | Species (*P value*) | Drought (*Df*) | Drought (*F value*) | Drought (*P value*) | Species × Drought (*Df*) | Species × Drought (*F value*) | Species × Drought (*P value*) | $R^2$ |
|---|---|---|---|---|---|---|---|---|---|---|---|
| All species | Soluble sugars | 7 | 38.2 | **<0.01** | 1 | 0.02 | 0.891 | 7 | 1.97 | 0.09 | 0.88 |
| | Starch | 7 | 28.9 | **<0.01** | 1 | 55.4 | **<0.01** | 7 | 8.4 | **<0.01** | 0.89 |
| | NSCs | 7 | 30.4 | **<0.01** | 1 | 18.9 | **<0.01** | 7 | 3.1 | **0.01** | 0.87 |
| Canopy species | Soluble sugars | 2 | 6.1 | **0.01** | 1 | 0 | 0.97 | 2 | 3.4 | **0.06** | 0.54 |
| | Starch | 2 | 9.9 | **<0.01** | 1 | 16.5 | **<0.01** | 2 | 5.3 | **0.02** | 0.75 |
| | NSCs | 2 | 3.5 | **0.05** | 1 | 1.5 | 0.24 | 2 | 0.8 | 0.47 | 0.39 |
| Understory species | Soluble sugars | 4 | 135.9 | **<0.01** | 1 | 0.1 | 0.74 | 4 | 1 | 0.42 | 0.96 |
| | Starch | 4 | 27 | **<0.01** | 1 | 39.7 | **<0.01** | 4 | 8 | **<0.01** | 0.89 |
| | NSCs | 4 | 57.9 | **<0.01** | 1 | 25.8 | **<0.01** | 4 | 5.3 | **<0.01** | 0.93 |

Numbers represent p-values. ANOVA was conducted with Type I sums of squares. NSCs, nonstructural carbohydrates.

**Table S4.** Two-way ANOVA testing effects of drought and species and their interactions on the amount of the $^{13}C$ label.

| Species | Pools | Species (*Df*) | Species (*F value*) | Species (*P value*) | Drought (*Df*) | Drought (*F value*) | Drought (*P value*) | Species × Drought (*Df*) | Species × Drought (*F value*) | Species × Drought (*P value*) | $R^2$ |
|---|---|---|---|---|---|---|---|---|---|---|---|
| All species | Leaf excess $^{13}C$ | 7 | 9.6 | **<0.01** | 1 | 18.3 | **<0.01** | 7 | 2 | 0.08 | 0.72 |
| Canopy species | Leaf excess $^{13}C$ | 2 | 2.6 | 0.11 | 1 | 8.1 | **0.01** | 2 | 2.5 | 0.11 | 0.53 |
| | Phloem excess $^{13}C$ | 2 | 0.03 | **0.97** | 1 | 9.8 | **<0.01** | 2 | 0.3 | 0.77 | 0.39 |
| Understory species | Leaf excess $^{13}C$ | 4 | 15.7 | **<0.01** | 1 | 10.2 | **<0.01** | 4 | 2.2 | 0.1 | 0.79 |
| | Root excess $^{13}C$ | 4 | 25 | **<0.01** | 1 | 7.1 | **0.02** | 4 | 17 | **<0.01** | 0.91 |

Numbers represent p-values. ANOVA was conducted with Type I sums of squares. The $^{13}C$ label represents the excess $^{13}C$ relative to the pre-labeling $^{13}C$ values in atom % in leaves (at day 0), stem phloem (at day 4) and roots (at day 4). The amount of excess $^{13}C$ (atom %) in leaves after labeling under drought was divided by 2.1 prior to test to account for the differences in excess $^{13}C$ in the atmosphere before (1.30 atom %) and during drought (2.73 atom %) (Werner et al., 2021).

**Table S5.** Correlation coefficient (R) and significance (P) of the linear regressions (see Fig. S2) of leaf sugars, starch, total NSCs versus time for the three canopy species.

| | | Sugars | | Starch | | Total NSCs | |
|---|---|---|---|---|---|---|---|
| Position | Species | *R* | *P* | *R* | *P* | *R* | *P* |
| Leaf | *Clitoria fairchildiana* | **0.74** | **< 0.01** | **−0.66** | **< 0.05** | 0.05 | 0.88 |
| | *Phytolacca dioica* | −0.21 | 0.53 | −0.26 | 0.44 | −0.24 | 0.47 |
| | *Pachira aquatica* | **−0.82** | **< 0.01** | **−0.83** | **< 0.01** | **−0.83** | **< 0.01** |
| Stem | *Clitoria fairchildiana* | **0.84** | **< 0.01** | −0.09 | 0.84 | 0.64 | 0.09 |
| | *Phytolacca dioica* | 0.41 | 0.31 | - | - | 0.41 | 0.31 |
| | *Pachira aquatica* | 0.61 | 0.15 | 0.24 | 0.61 | 0.61 | 0.15 |

NSCs, nonstructural carbohydrates.

**Table S6.** Correlation coefficient (R) and significance (P) of the linear regressions (see Fig. S3) of leaf sugars, starch, total NSCs versus time for the five understory species.

| | | Sugars | | Starch | | Total NSCs | |
|---|---|---|---|---|---|---|---|
| Position | Species | *R* | *P* | *R* | *P* | *R* | *P* |
| Leaf | *Piper auritum* | −0.05 | 0.89 | **−0.79** | **< 0.01** | **−0.69** | **0.03** |
| | *Hibiscus rosa sinensis* | −0.18 | 0.61 | **−0.82** | **< 0.01** | **−0.79** | **< 0.01** |
| | *Calathea sp.* | 0.48 | 0.16 | 0.06 | 0.88 | 0.56 | 0.09 |
| | *Syngonium sp.* | −0.31 | −0.31 | 0.14 | 0.69 | −0.24 | 0.51 |
| | *Diefenbachia sp.* | −0.21 | 0.56 | −0.54 | 0.11 | −0.58 | 0.08 |
| Root | *Piper auritum* | **0.97** | **< 0.05** | −0.71 | 0.29 | −0.15 | 0.85 |
| | *Hibiscus rosa sinensis* | 0.58 | 0.42 | **0.94** | **0.06** | 0.78 | 0.22 |
| | *Calathea sp.* | 0.66 | 0.34 | 0.67 | 0.34 | 0.66 | 0.34 |
| | *Syngonium sp.* | 0.21 | 0.79 | 0.04 | 0.96 | 0.11 | 0.89 |
| | *Diefenbachia sp.* | 0.84 | 0.16 | −0.59 | 0.41 | 0.78 | 0.22 |

NSCs, nonstructural carbohydrates.

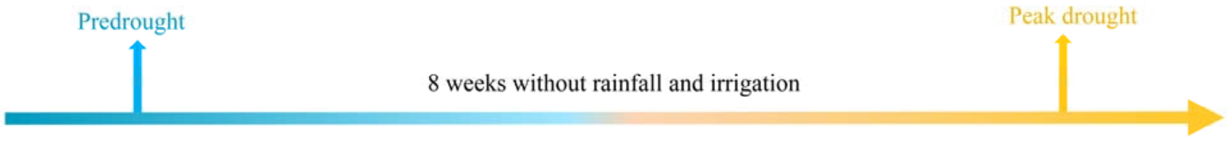


First $^{13}CO_2$ labeling campaign

| Group | Leaf | Phloem | Root |
|---|---|---|---|
| Canopy species | -1, 0, 1, 3, 5 | -1, 3, 4, 9 | -1, 4 |
| Understory species | -1, 0, 1, 3, 5 | NA | -1, 3 |

Second $^{13}CO_2$ labeling campaign

| Group | Leaf | Phloem | Root |
|---|---|---|---|
| Canopy species | -1, 0, 1, 3, 5 | -1, 4, 8 | -1, 4* |
| Understory species | -1, 0, 1, 3, 5 | NA | -1, 4 |

Unit: Days after the labeling

**Fig. S1.** The drought treatment, labeling and sampling timeline. To impose drought treatment, rainfall and irrigation were withheld from 8 Oct, 2019 to 2 December 2019 (c. 8 weeks). We conduced $^{13}CO_2$-pulse labeling under pre-drought conditions (5 Oct, 2019; left table) and under drought conditions (23 Nov, 2019; right table). Samples were collected for NSC analysis and isotopic tracing during each labeling campaign: 1) leaves were sampled before labeling and 0, 1, 3 and 5 days after labeling; 2) stem phloem samples from the canopy trees were collected before labeling, and 3 to 9 days after labeling; 3) roots were collected before labeling, and 3 to 4 days after the labeling. Samples were immediately frozen in liquid nitrogen or dry ice to stop metabolic activity, and stored at -20 °C. Note that post-labeling root samples collected from the canopy trees under drought were lost during shipping process and not available for analyses. All samples were transported on dry ice by car, freeze-dried, and ground to fine powder before metabolite and isotope analysis.

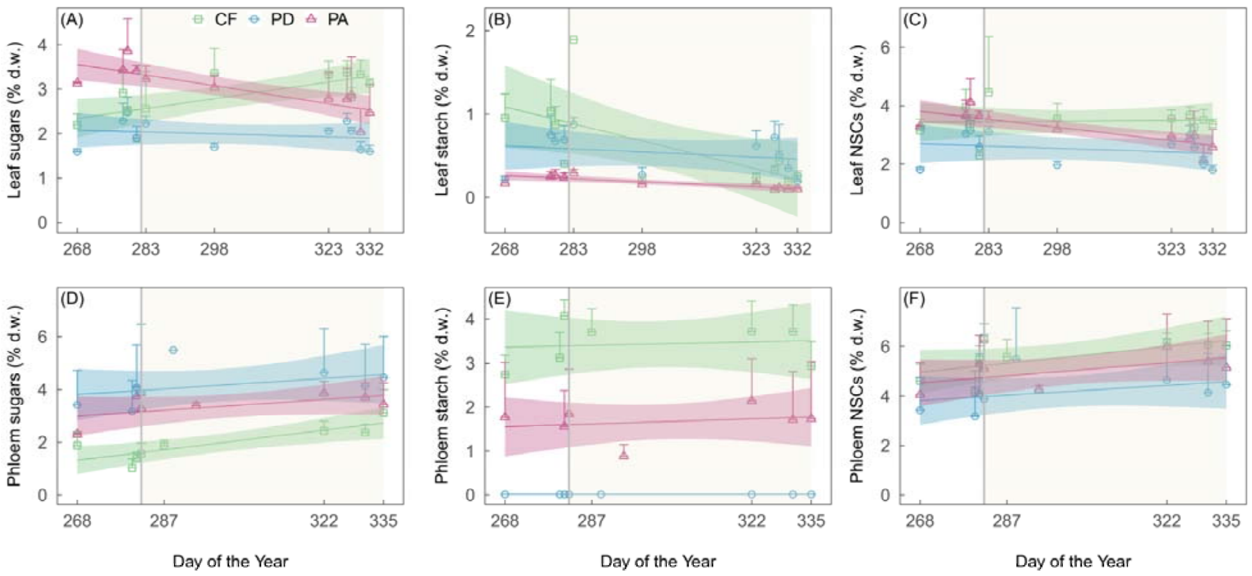


**Fig. S2.** Changes in concentrations of soluble sugars, starch, and total nonstructural carbohydrates (NSCs; soluble sugars + starch) in the leaves and stem phloem over the course of the experiment in the three canopy tree species: *Clitoria fairchildiana* (CF), *Phytolacca dioica* (PD), *Pachira aquatica* (PA). Values are the means of 3 or 4 plants per species, and error bars represent standard errors. Background shadings indicate the drought days. See Table S5 for correlation coefficient (R) and significance (P).

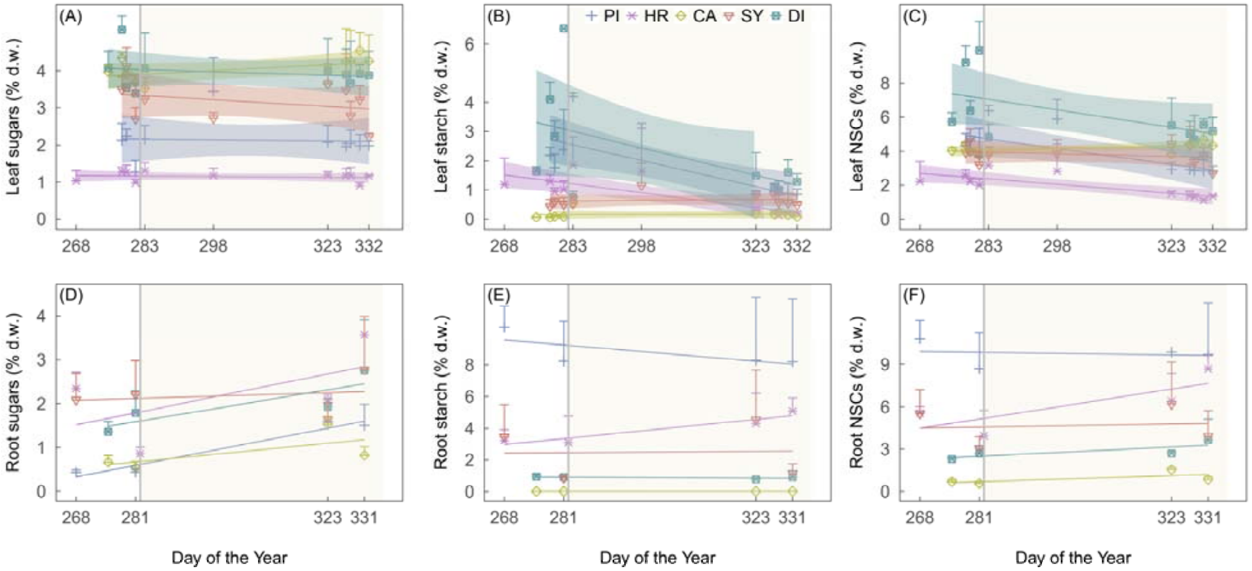


**Fig. S3.** Changes in concentrations of soluble sugars, starch, and total nonstructural carbohydrates (NSCs; soluble sugars + starch) in the leaves and roots over the course of the experiment in the five understory tree species: *Piper auritum* (PI), *Hibiscus rosa sinensis* (HR), *Calathea* sp. (CA), *Syngonium* sp. (SY), *Dieffenbachia* sp. (DI). Values are the means of 3 or 4 plants per species, and error bars represent standard errors. Background shadings indicate the drought days. See Table S6 for correlation coefficient (R) and significance (P).

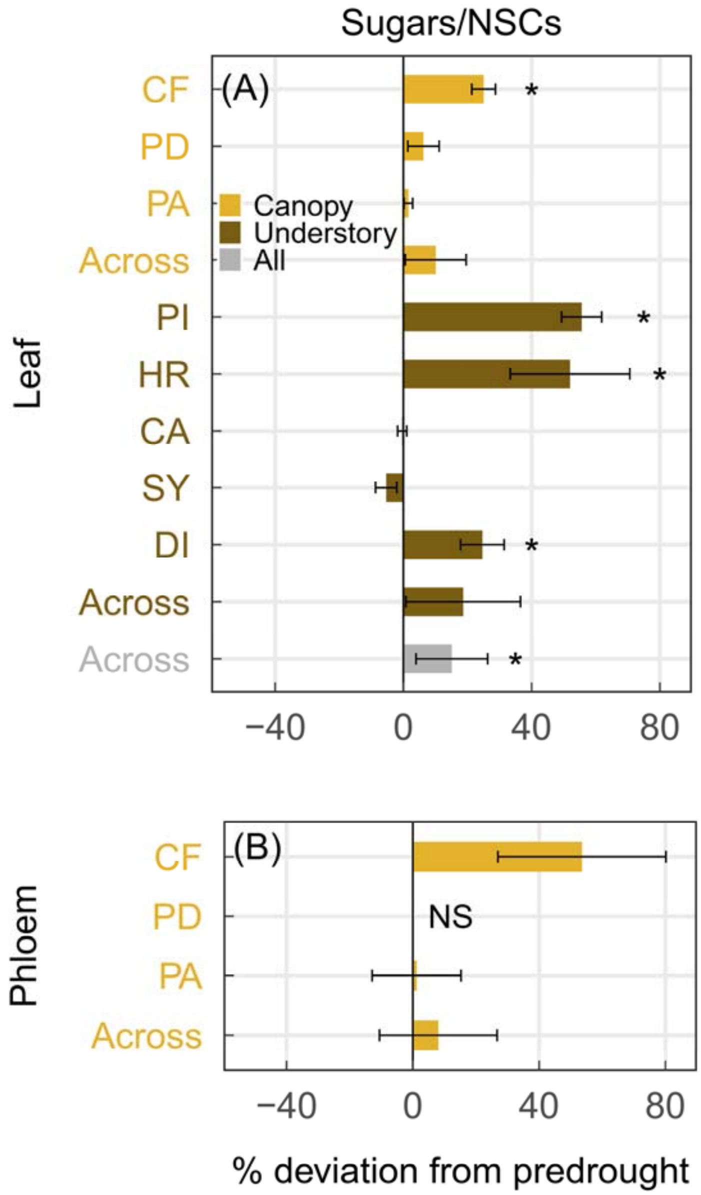


**Fig. S4.** Relative changes in the ratio of soluble sugars to nonstructural carbohydrates (sugars/NSCs) in the leaves (a) and stem phloem (b) under drought, expressed as percent deviations from pre-drought values (n = 3 or 4 plants per species). Data are shown for the three canopy species including *Clitoria fairchildiana* (CF), *Phytolacca dioica* (PD), *Pachira aquatica* (PA), and five understory species including *Piper auritum* (PI), *Hibiscus rosa sinensis* (HR), *Calathea* sp. (CA), *Syngonium* sp. (SY), *Diefenbachia* sp. (DI), as well as averaged (AVG) across the species means (grey). Percent deviations are not computed (NS) for PD phloem due to low starch concentrations (<0.5%; Fig. S2). Positive values represent increase under drought and negative values represent decrease. Error bars represent standard errors. Significant within-species (Student's t-test) and cross-species (two-way ANOVA) differences between pre-drought and drought were calculated based on the raw concentrations and indicated by an asterisk ($P < 0.05$).

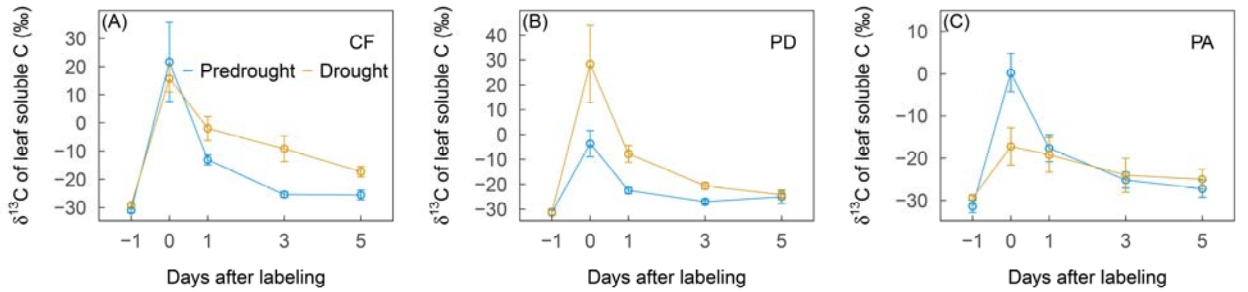


**Fig. S5.** Changes in δ$^{13}$C of leaf soluble carbon for the three canopy species (*Clitoria fairchildiana*, CF; *Phytolacca dioica*, PD; *Pachira aquatica*, PA) under pre-drought and drought conditions (n = 3 or 4 plants per species). Values are the means of 3 or 4 plants per species, and error bars represent standard errors. Note that we added twice as much $^{13}CO_2$ label to the atmosphere to compensate for the reduction in photosynthesis under drought.

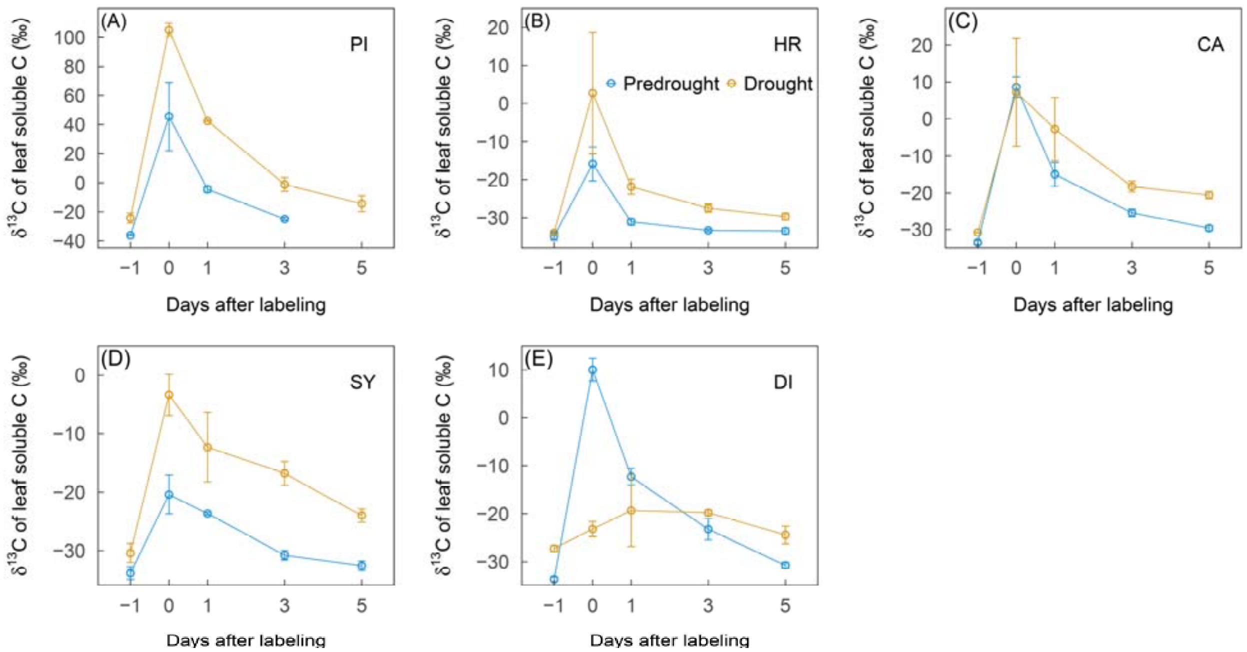


**Fig. S6.** Changes in δ$^{13}$C of leaf soluble carbon for the five understory species (*Piper auritum*, PI; *Hibiscus rosa sinensis,* HR; *Calathea* sp., CA; *Syngonium* sp., SY; *Dieffenbachia* sp., DI) under pre-drought and drought conditions (n = 3 or 4 plants per species). Values are the means of 3 or 4 plants per species, and error bars represent standard errors. Note that we added twice as much $^{13}CO_2$ label to the atmosphere to compensate for the reduction in photosynthesis under drought.

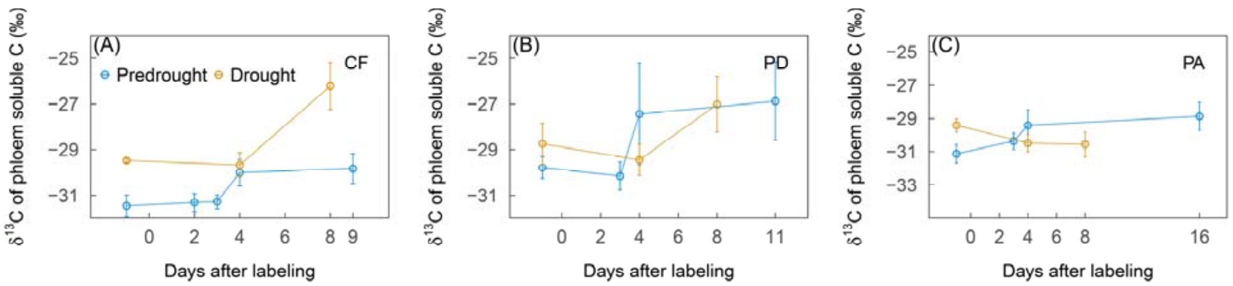


**Fig. S7.** Changes in δ$^{13}$C of phloem soluble carbon for the three canopy species (*Clitoria fairchildiana*, CF; *Phytolacca dioica*, PD; *Pachira aquatica*, PA) under pre-drought and drought conditions (n = 3 or 4 plants per species). Values are the means of 3 or 4 plants per species, and error bars represent standard errors.

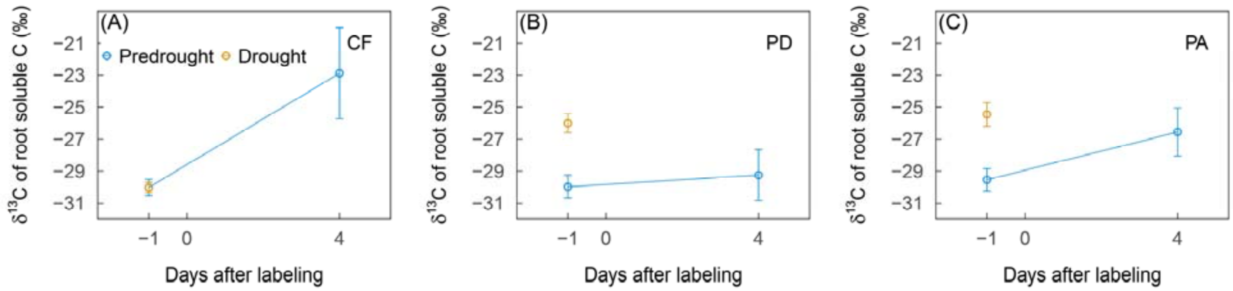


**Fig. S8.** Changes in $\delta^{13}C$ of root soluble carbon for the three canopy species (*Clitoria fairchildiana*, CF; *Phytolacca dioica*, PD; *Pachira aquatica*, PA) under pre-drought and drought conditions (n = 3 or 4 plants per species). Values are the means of 3 or 4 plants per species, and error bars represent standard errors.

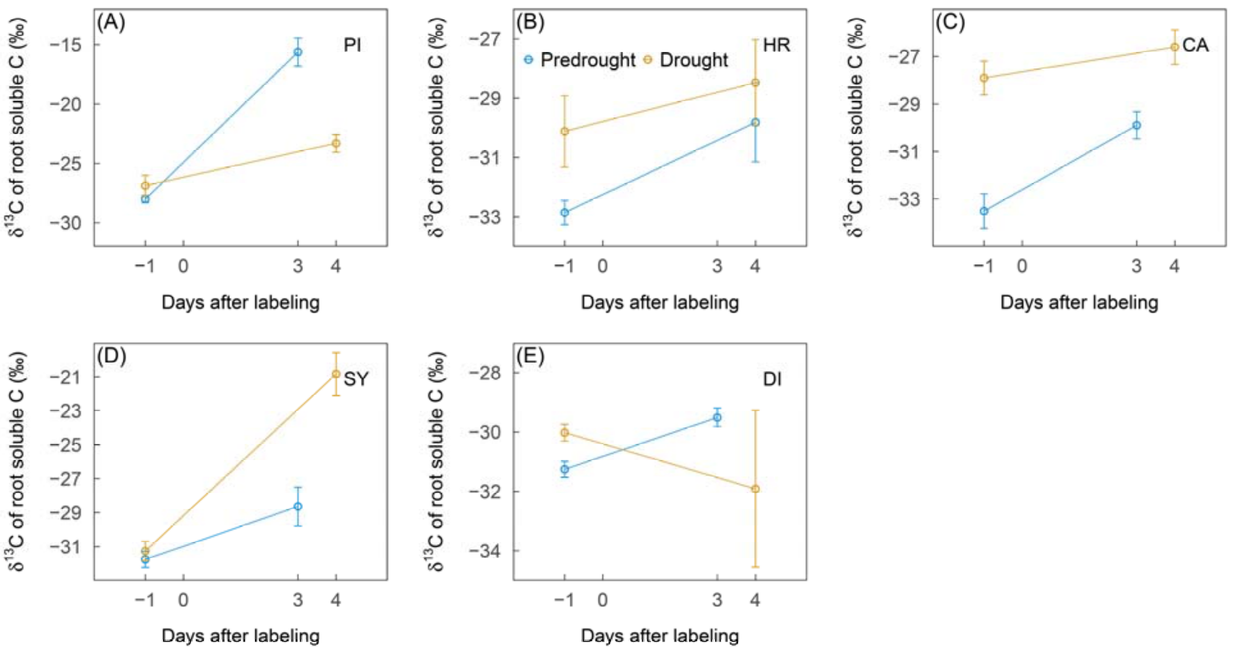


**Fig. S9.** Changes in $\delta^{13}C$ of root soluble carbon for the five understory species (*Piper auritum*, PI; *Hibiscus rosa sinensis,* HR*; Calathea* sp.*,* CA; *Syngonium* sp.*,* SY; *Dieffenbachia* sp., DI) under pre-drought and drought conditions (n = 3 or 4 plants per species). Values are the means of 3 or 4 plants per species, and error bars represent standard errors.

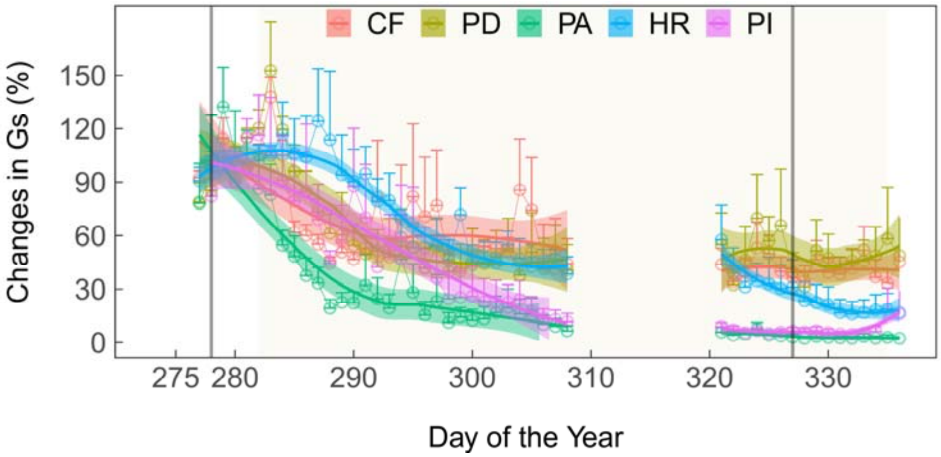


**Fig. S10.** Changes in stomatal conductance (Gs) over the course of the experiment in five species: *Clitoria fairchildiana* (CF), *Phytolacca dioica* (PD), *Pachira aquatica* (PA), *Piper auritum* (PI)*, Hibiscus rosa sinensis* (HR). Values are the means of 3 or 4 plants per species, expressed as a percentage of pre-drought. Error bars represent standard errors. Background shadings indicate the drought days. Grey lines indicate the pulse-labeling events.

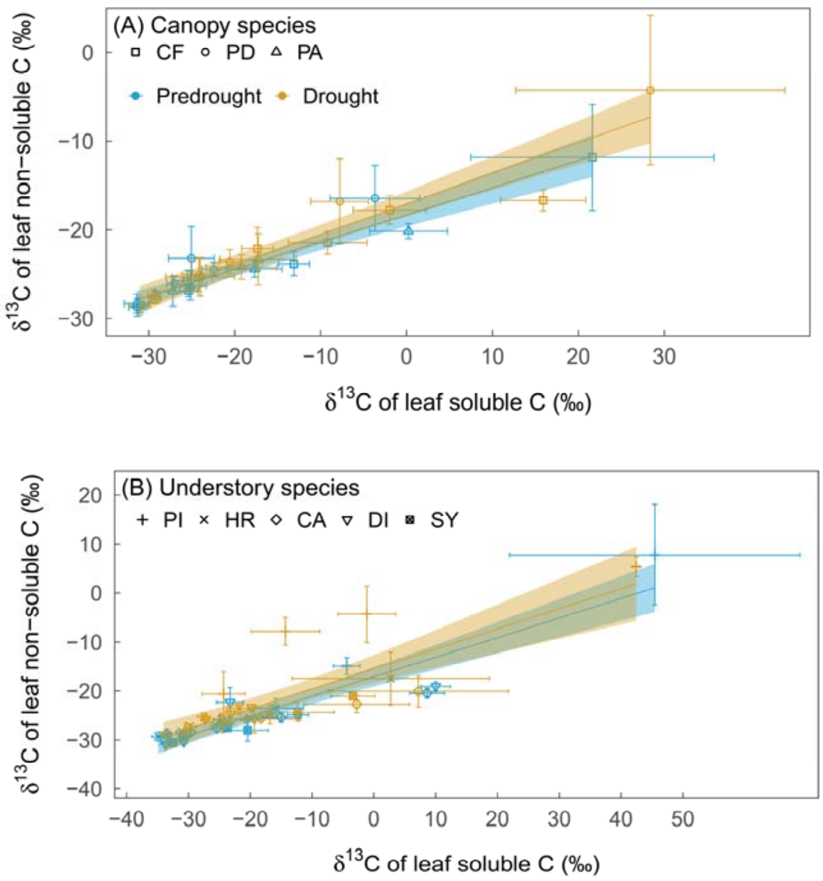


**Fig. S11.** Changes in $\delta^{13}C$ of leaf soluble carbon versus non-soluble carbon for the three canopy species (*Clitoria fairchildiana,* CF; *Phytolacca dioica*, PD; *Pachira aquatica,* PA) and five understory species (*Piper auritum*, PI; *Hibiscus rosa sinensis,* HR*; Calathea* sp*.,* CA; *Syngonium* sp*.,* SY; *Dieffenbachia* sp*.*, DI) under pre-drought and drought conditions (n = 3 or 4 plants per species). See Method S3 for details. Values are the means of 3 or 4 plants per species, and error bars represent standard errors. Across canopy and understory species, there were significant correlations and these correlations were not affected by drought, indicating that drought did not affect partitioning of recent photosynthates into soluble vs. non-soluble carbon pools.

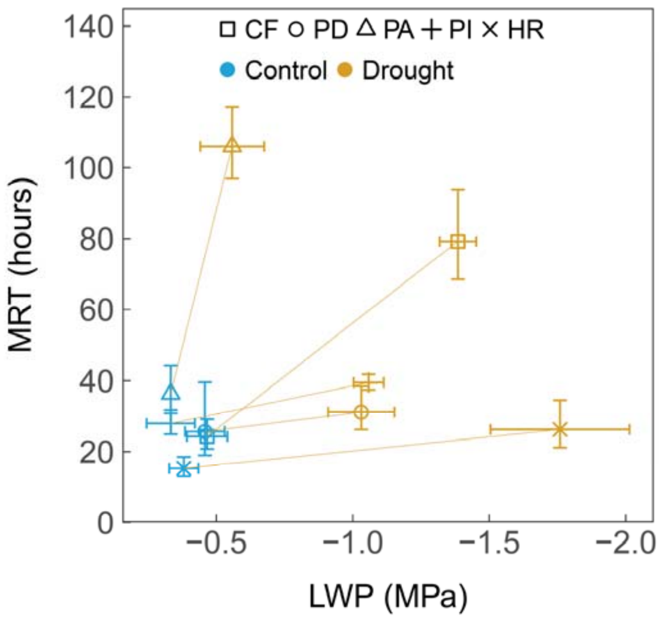


**Fig. S12.** The relationships between midday leaf water potential and the absolute MRT (hours) of the $^{13}C$ label in leaf soluble carbon for the three canopy species (*Clitoria fairchildiana,* CF; *Phytolacca dioica*, PD; *Pachira aquatica,* PA) and two understory species (*Piper auritum*, PI; *Hibiscus rosa sinensis,* HR) under pre-drought (blue) and drought (yellow) conditions (n = 3 or 4 plants per species).

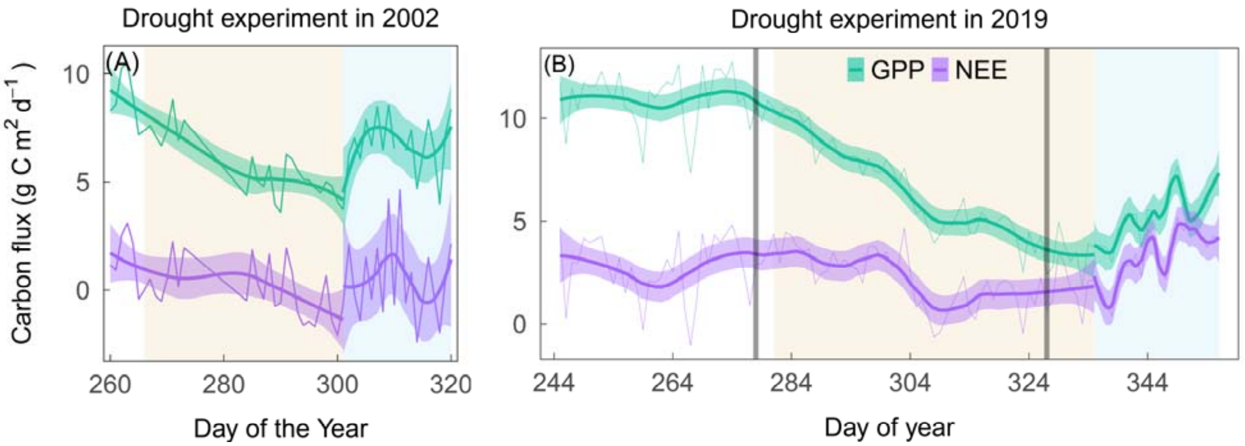


**Fig. S13.** Changes in gross primary productivity (GPP) during the imposed drought (yellow background shading) and recovery (blue background shading) in 2002 (left panel) and 2019 (right panel). Grey lines indicate the pulse-labeling period in the 2019 experiment. In both experiments, GPP decreased during drought and then increased after re-watering.

**References**


Werner, C., Meredith, L. K., Ladd, S. N., Ingrisch, J., Kubert, A., van Haren, J., . . . Williams, J. (2021). Ecosystem fluxes during drought and recovery in an experimental forest. *Science, 374*(6574), 1514-1518. doi:10.1126/science.abj6789
